# Fluorimetric microplate assay for the determination of extracellular alkaline phosphatase kinetics and inhibition kinetics in activated sludge

**DOI:** 10.1016/j.mex.2023.102255

**Published:** 2023-06-15

**Authors:** Klaus Fischer, Marion Wacht

**Affiliations:** Analytical and Ecological Chemistry, Faculty VI, University of Trier, Behringstr. 21, 54296 Trier, Germany

**Keywords:** Enzyme kinetics, Enzyme inhibition, Enzymatic reactions, Biological wastewater treatment, Organic phosphorus compounds, Phosphor elimination, Fluorimetric microplate assay for the determination of extracellular alkaline phosphatase kinetics and inhibition kinetics in activated sludge

## Abstract

The microbial enzyme alkaline phosphatase contributes to the removal of organic phosphorus compounds from wastewaters. To cope with regulatory threshold values for permitted maximum phosphor concentrations in treated wastewaters, a high activity of this enzyme in the biological treatment stage, e.g., the activated sludge process, is required. To investigate the reaction dynamics of this enzyme, to analyze substrate selectivities, and to identify potential inhibitors, the determination of enzyme kinetics is necessary. A method based on the application of the synthetic fluorogenic substrate 4-methylumbelliferyl phosphate is proven for soils, but not for activated sludges. Here, we adapt this procedure to the latter. The adapted method offers the additional benefit to determine inhibition kinetics. In contrast to conventional photometric assays, no particle removal, e.g., of sludge pellets, is required enabling the analysis of the whole sludge suspension as well as of specific sludge fractions. The high sensitivity of fluorescence detection allows the selection of a wide substrate concentration range for sound modeling of kinetic functions.

• Fluorescence array technique for fast and sensitive analysis of high sample numbers
• No need for particle separation – analysis of the whole (diluted) sludge suspension
• Simultaneous determination of standard and inhibition kinetics

## Graphical abstract

**Figure:**
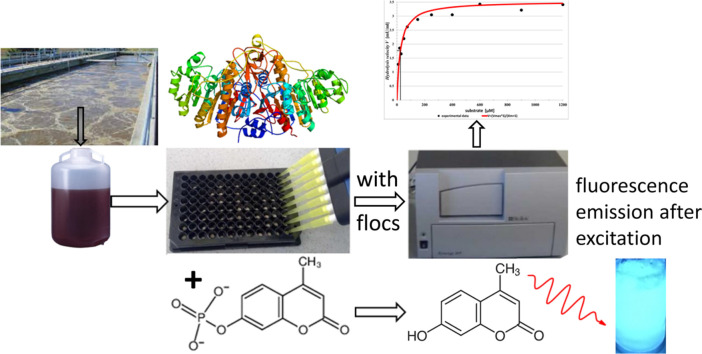

Specifications table

**Table:** 

Subject area:	Biochemistry, Genetics and Molecular Biology
More specific subject area:	Environmental Enzymology
Name of your method:	Fluorimetric microplate assay for the determination of extracellular alkaline phosphatase kinetics and inhibition kinetics in activated sludge
Name and reference of original method:	Marx, M.-C., Wood, M., Jarvis, S.C., A microplate fluorimetric assay for the study of enzyme diversity in soils. Soil Biol. Biochem. 33 (2001) 1633-1640.
Resource availability:	*N/A*

## Method details

### Background

Since enzymes are the decisive microbial tools to degrade and eliminate the organic wastewater load, knowledge of their reactivity, e.g., substrate selectivity and turn-over rates, is crucial for the maintenance of a high process efficiency of biological wastewater treatment units, e.g. activated sludge reactors. Most enzymological studies in wastewater treatment plants (WWTP) are restricted to the measurement of enzyme activities, leaving the potential of in-depth kinetic information unexploited. As far as enzyme kinetics were determined in activated sludge, a photometric method was applied [1], afflicted with several drawbacks, i.e., limitation to low sample numbers, low sensitivity, need for particle removal and endpoint measurement instead of continuous monitoring of the reaction progress. These drawbacks could be avoided by the adaptation of a microplate fluorimetric assay, which is “state-of-the-art” in soil enzymology but unproven for activated sludge [2]. To solve this task, we focused on the enzyme “alkaline phosphatase” (APA), but due to the systematic commonalities of fluorimetric methods, based on the analysis of the same fluorogenic label, the method should also be applicable – with a few modifications – for other hydrolases, in so far as 4-methylumbelliferyl (4-MUF) labelled substrates are available. Expanding the scope of the original method, inhibition kinetics were included additionally.

### Reagents and solutions

- Methanol, HPLC-MS grade (VWR, P/N 83638.320), used for the preparation of the 4-MUF stock solution.
- HEPES (2-[4-(2-hydroxyethyl)-1-piperazinyl] ethane sulfonic acid), high purity free acid (VWR, P/N 0511-1kg), used for the preparation of aqueous buffer solutions. Buffer concentration: 0.05m (dissolve 11.916 g of HEPES in 1L of water). The buffer pH value is adjusted to 7.50 by addition of diluted NaOH solution.
- NaOH solution, 50% (weight), for analysis (Merck, P/N 1.58793.1000)
- 4-Methylumbelliferone (4-MUF, IUPAC name: 7-Hydroxy-4-methylchromen-2-one) (Aldrich, P/N M1381-25g), fluorogenic label, used for the calibration of the enzymatic hydrolysis of the substrate 4-MUF phosphate (4-MUF as a reaction product).
  Stock solution: Dissolve 0.1 millimole (17.6 mg) in 10 mL of methanol. This 10 mM stock solution, stored at 4°C in a refrigerator, is stable over one month at least. Working solutions (0.1 mM, 10.0 µM, 1.0 µM) were weekly prepared by dilution of corresponding aliquots of the stock solution or further diluted solutions by addition of the required HEPES buffer volumes.
- 4-Methylumbelliferyl phosphate (4-MUF-P) (Biosynth-Carbosynth, P/N EM09307): APA substrate.
  Stock solution, freshly prepared every day: Dissolve 0.1 millimole (25.6 mg) in 10 mL of HEPES solution. Working solutions were prepared as described above for 4-MUF.
- Sodium tungstate dihydrate for analysis (Merck, P/N 3373 100g), applied for the determination of inhibition kinetics. Prepare a 1.0 mM stock solution by dissolution of 329.8 mg of Na_2_WO_4_ _*_ 2 H_2_O in 100 mL of HEPES buffer solution. Dilute aliquots further in a ratio 1:10 by mixing with appropriate HEPES volumes. Store the solutions at 4°C in a refrigerator.

### Materials

- 96-Well black flat-bottom microplates (Thermo Scientific, P/N 237108)
- Graduated screw tubes with pointed bottom, 15 and 50 ml (Sarstedt, P/N 62.554.502 and 22.547.254)
- Universal pipette tips, various sizes (VWR)

### Instrumentation/apparatus

- Astacus reagent ultrapure water system (P/N 110-000, membraPure, Henningsdorf, Germany)
- 8-Channel pipette, variable volumes (20 - 200µL) (P/N 613-5252, VWR, Darmstadt, Germany), for reagent and sample dosage into the plate wells
- End-over-end shaker (REAX2, Heidolph, Berlin, Germany) for coarse dispersion of the sludge samples
- Ultra-sonication bath (BANDELIN Electronic, Germany), frequency 35 kHz, operated at 70% of the maximum energy output, for the fine dispersion and disintegration of activated sludge flocs.
- Synergy HT plate reader (Bio-Tek Instruments, Bad Reichenhall, Germany), controlled by the Gen5 software (Bio-Tek Instruments), for microplate reading of the 4-MUF fluorescence at excitation wavelength of 360 nm and emission wavelength of 460 nm. Gain settings (70 – 60) depended on sludge dilution, substrate concentration and recording period. Main fixed operation parameters were: top optics position with a top probe vertical offset of 1.00 mm, normal read speed, and thermostatic control of the plates at 30°C.

### Protocol

**Fig. 1:**
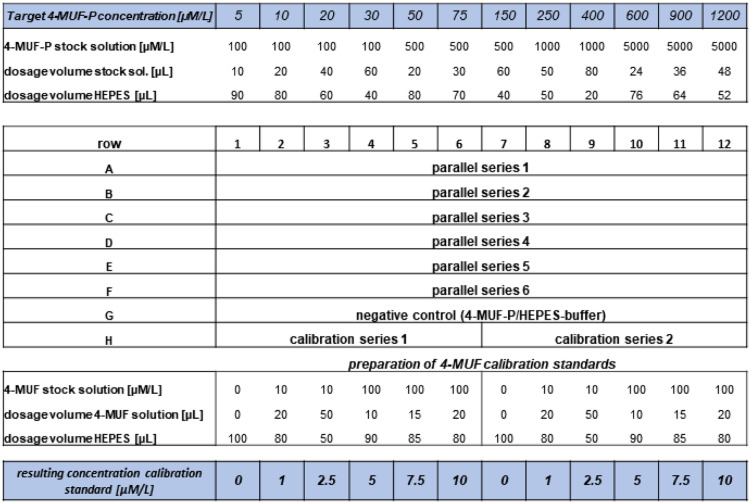
Microplate pipette scheme for the determination of APA standard kinetics. All wells except those of the negative control (row G) receive 100 µL of prediluted activated sludge suspension. The volumes of the 4-MUF-P or of the 4-MUF stock solutions and that of the HEPES solutions sum up to 100 µL always, resulting in a total well filling volume of 200 µL including the sludge suspension.
  11) Without delay, add quickly 100 µL of the various substrate solutions, preheated to 30°C, to the wells of the remaining rows filled with activated sludge.
    A microplate lay-out for the determination of standard kinetics is displayed in Fig. 1.
  12) Immediately place the plate into the sample chamber of the reader and start the measuring program. *Note: A typical kinetic program lasts 15 min with fluorescence reading every 90 s.*
  13) Transformation of the analytical raw data (RFU: relative fluorescence units) into 4-MUF amounts per well:
    - Transfer the raw data from the plate reader into a MS Excel sheet or into a corresponding spreadsheet program.
    - Calculate means both for the parallels of the kinetic tests (sample data) and for the two calibrations sets. *Note: Additional calculation of the coefficients of variation (relative standard deviation) of the kinetic test replicates provides an indicator for the homogeneity of the enzymatic activities of the sludge aliquots, for potential outliers and for operational errors.*
    - Subtract the RFU values of the negative controls from the means of the test replicates with identical initial substrate concentrations for every measuring time.
    - Calculate a linear calibration function based on the means of the duplicates of the equally concentrated calibration standards for each measurement interval separately. Convert 4-MUF concentrations [µM/L] into 4-MUF amounts [nmol/well] by division with the factor 5.
    - Convert the sample RFU values into 4-MUF amounts per well by applying the simultaneously recorded calibration functions.
    - Subtract the apparent 4-MUF amount of the first reading (time “zero”) from all further readings of the same well (setting the first value to zero). In the case of a negative value, add it to all readings. *Note: this step accounts for an under- or overcompensation by the negative controls and for the 4-MUF generation during the period between substrate dosage and first reader record.*
  14) Convert the corrected “4-MUF/well” data into “4-MUF/mL original sludge suspension” data by multiplying the former with the sludge dilution factor and with the reciprocal of the dosed sludge volumes in the wells, given in the unit “mL”. *Note: for instance, a dilution factor of 5 and a sludge dosage volume of 0.1 mL will result in an overall factor of 50.*
  15) Calculate the enzymatic reaction rate for every specific initial substrate concentration by linear regression of the generated 4-MUF amounts with time. The unit [nmol/mL*min] equals [mU/mL], where mU is a conventional unit for enzyme activity. Check the coefficients of determination (r^2^) for the match between recorded data and modelled kinetic functions, i.e., for deviations from linearity.
  16) Calculate the Michaelis-Menten kinetic function for the overall reaction by nonlinear regression of the reaction rates from step 15 with the initial substrate concentrations. This calculation delivers the Michaelis-Menten constant K_M_ (substrate concentration for half-maximal reaction velocity) and the maximal reaction velocity V_max_. If MS Excel is used for data treatment, activate, and run the “Solver” add-in to perform the nonlinear regression analysis.
B) Modification of the method for the determination of inhibition kinetics
  1) General notes
    - Applied inhibitors must not exhibit auto-fluorescence in the fluorescence emission range of the fluorogenic label, i.e., 4-MUF. Specific requirements stem from the wavelength resolution of the plate reader.
    - Check in advance potential alterations of the 4-MUF-P and 4-MUF fluorescence by interactions with the inhibitor.
    - Depending on the chemical properties of the inhibitor, check solubility as a function of the pH value.
  2) Modifications
    - step 7 and 10: To control and compensate potential fluorescence alterations by the inhibitor most precisely, the selected inhibitor concentration should be constituted in the negative controls and in the calibration standards, e.g., by addition of 50 µL of the fourfold concentrated inhibitor solution into the wells. If two test series with different inhibitor concentrations are to be conducted on one plate, adjust the mean inhibitor concentration in the controls. Modify the substrate and the 4-MUF dosage concentrations with respect to a dosage volume of 50 µL. *Note: In cases, where preceding tests have already proven that the inhibitor does not significantly alter the fluorescence properties of the samples and of the reference compounds, this modification is not strictly required.*
    - step 11: Immediately before substrate addition to the wells, transfer 50 µL of the inhibitor solutions to them. Double the substrate concentration with respect to the half dosage volume (50 µL).
    - Microplate pipette schemes.
      To determine the specific inhibition mechanism, enzymatic substrate hydrolysis has to be measured in the absence and in the presence (three different concentrations at least) of the inhibitor under otherwise identical reaction conditions. Since three parallels are required for every test condition to achieve statistically sound data, one complete kinetic experiment consumes two plates at least.
      The pipette maps might look as follows:
      plate 1:
      rows A-C: activated sludge plus substrate – no inhibitor added
      rows D-F: activated sludge plus substrate plus inhibitor concentration “X”, e.g., 5 µM tungstate
      row G: negative controls
      row H: Wells 1-6: calibration standards without inhibitor addition; wells 7-12: with adjusted inhibitor concentration “X”
      plate 2:
      rows A-C: activated sludge plus substrate and inhibitor concentration “Y”, e.g., 25 µM tungstate
      rows D-F: as rows A-C, inhibitor concentration “Z”, e.g. 100 µM tungstate
      row G: negative controls
      row H: wells 1-6: calibration standards containing inhibitor concentration “Y”, wells 7-12: standards with inhibitor concentration “Z”
    An example of a plate lay-out testing two inhibitor concentrations simultaneously is depicted in Fig. 2.

A) APA kinetic assay
  1) Sample the activated sludge from an aerated sludge tank of a wastewater treatment plant by submersion of a precleaned HDPE canister and transport it subsequently to the research laboratory. If not immediately used, store it in a refrigerator. *Note: Due to the ongoing change of the microbial activity and composition of activated sludge samples they should be further processed at sampling day. If storage over several hours is required, maintain the biological activity by supply with air and nutrients. Activated sludge is a potential pathogenic material. Persons working with it must wear adequate protective equipment and should have been vaccinated against hepatitis b. Clean benches and other laboratory equipment with biocidal solutions, e.g., ethanol.*
  2) Immediately before further use, resuspend the sludge by agitation in an end-over-end shaker or in a similar device during 5 min.
  3) Constitute a subsample with a volume of 50 mL by pouring of an aliquot into a screw tube.
  4) Insert the screw tube into the ultrasonic bath and treat it during 10 min at 70% of the maximum energy output to widely disperse the flocs, transferring occluded or attached enzymes into the solution without disruption of the microbial cell walls (discrimination between extracellular and intracellular APA fraction). *Note: The suited operational conditions depend on the specific instrumental properties. To identify these conditions, the potential release of intracellular enzymes should be controlled by measuring the activity of dehydrogenase, an exclusively intracellular enzyme, e.g., according to a German analytical norm* [3]*. Conduct a test series varying ultrasonic treatment periods and radiation energies. Optimal conditions are characterized by highest APA activity at bottom level of DHA activity.*
  5) Dilute an aliquot of the dispersed sludge by addition of the required volume of the HEPES solution. *Note: Typical dilution ratios range from 1:5 to 1:10. For instance, to fill every well of a 96-well plate with 100 µL of a 1:5 diluted sludge sample, 1.92 mL of the original sludge suspension are needed. The dilution ratio depends on the sludge dry matter content, the APA activity, and the required analytical sensitivity at selected time resolution. The lower limit results from a reduced analytical signal reproducibility due to enhanced matrix effects (attenuation of excitation radiation, quenching and reflection of emission radiation by particles or flocs). The upper dilution limit mainly stems from the loss of analytical sensitivity.*
  6) Select the 4-MUF phosphate concentrations to be applied in the kinetic tests. Note: *With respect to the number of wells within one row and with the purpose to generate a sufficient number of input data for proper kinetic modelling, 12 different substrate concentrations should be selected, stretching from the lower µM range up to about 1mM.*
  7) Negative controls: Constitute the substrate test concentrations in HEPES buffer by mixing of the respective aliquots of the diluted 4-MUF phosphate stock solution with corresponding volumes of the buffer solution. Fill one plate row with the negative controls. Total well filling volumes are 200 µL always.
  8) Fill each of the wells of at least 4 rows with 100 µL of the diluted activated sludge suspension by means of the 8-channel pipette. *Note: Check stability of the suspension and absence of settled flocs before filling of the plate.*
  9) Place the plate on the plate carrier, move it into the measurement chamber and warm it up to a temperature of 30°C during 10 min. *Note: To accelerate the reaction and to elevate the analytical sensitivity at low substrate concentration, the assay temperature was raised above the usual technical process temperature range. Since no information about the thermal stability of microbial extracellular APA in activated sludge suspensions is available, we do not recommend adjusting temperatures considerably higher than 30°C.*
  10) Remove the plate from the reader. Prepare two parallels of a set of 4-MUF calibration standards, comprising five 4-MUF concentrations plus blank, by addition of adequate buffer volumes, followed by the dosage of aligned volumes of 4-MUF stock solutions according to the pipette scheme shown in Fig. 1. Buffer and stock volumes sum up to 100 µL always. Add 100 µL HEPES solution to the blanks of the calibration sets. Finally, all wells are stocked up by addition of 100 µL of prediluted sludge suspension. *Note: A typical calibration range spans from 1.0 to 10.0 µM/L of 4-MUF, corresponding to 4-MUF amounts of 0.20 – 2.0 nmol per well.*

**Fig. 2:**
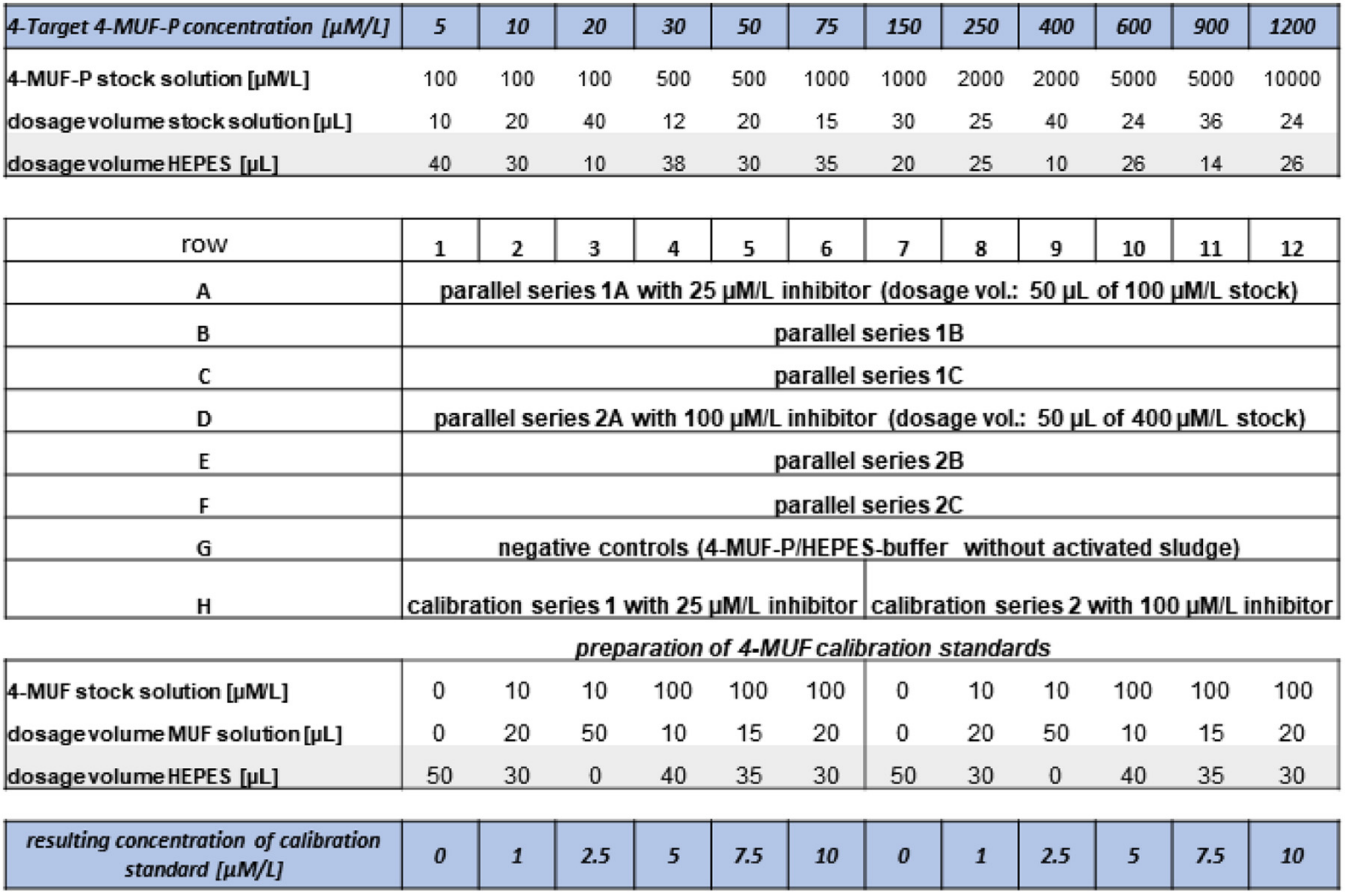
Microplate pipette scheme for the simultaneous determination of two inhibitor concentrations. Compared with standard kinetics (Fig. 1), doubled 4-MUF-P concentration in conjunction with half dosage volume (4-MUF-P stock solution plus HEPES). Calibration standards are composed of 50 µL of 4-MUF stock solution (including dosed HEPES) plus 50 µL of inhibitor solution plus 100 µL of prediluted sludge suspension.
  3) Evaluation and interpretation of the kinetic data

Calculate the Michaelis-Menten functions for the inhibition kinetics in the same way as for the standard kinetics (step 16). This will result in “apparent” K’_m_- and V’_max_-values. The various inhibition mechanisms differ in the dependence of these parameters on the inhibitor concentration. Indications for the inhibition type can be deduced from various plots of the partially transformed kinetic data or gained by the application of specific software packages, e.g., integrated in “GraphPad Prism” or “Sigma Plot”.

### Additional information

The developed method utilizes the benefits of fluorescence reading for the determination of enzyme kinetics in activated sludge. It enables the sensitive and almost continuous record of the reaction progress in the heterogenous sludge suspension with comparably low matrix interferences. Additionally, the costs of one standard kinetic assay are very low, i.e., a few Euros, summing up the proportional costs of the reagents and the consumables. The microplates are the major cost factor. Nevertheless, some critical process parameters have to be taken into account for the definition of optimized test conditions.

There are several reasons for the dilution of the sludge sample. A high particle density reduces the optical transparency of the suspension and might give rise to a significant quenching of the emitted fluorescence radiation as it is ascertained for soil suspensions [4]. The high APA content of the undiluted sludge would require the adjustment of high substrate concentrations to approximate enzyme saturation, which in turn would elevate the background fluorescence of the negative controls and potentially cause enzyme inhibition. Measuring inhibition kinetics, the inhibitory effect of the test compound might be regulated by its concentration ratio related to the substrate concentration. To prove this type of reaction dependence, high inhibitor concentrations are required, excluding sparingly soluble compounds as test candidates. Furthermore, lowering the enzyme concentration at fixed substrate concentration leads to an extension of the initial reaction interval where quasi steady-state conditions predominate (initial substrate concentration remains approximately constant), maintaining the conditions for a nearly constant hydrolysis rate.

On the other hand, dilution by addition of a buffer solution alters the composition of the water phase greatly with unknown consequences for enzyme reactivity. According to our experimental experiences, dilution ratios between 1 : 5 and 1 : 10 offer suitable test conditions. Analysing the kinetics of pure enzymes, the K_M_ values should be independent from enzyme concentration. Due to the heterogeneity of the sludge matrix and due to the different forms of interactions or associations between enzymes and sludge fractions, significant deviations from this “ideal behavior” are likely to occur. At least for APA, we did not found indications for a systematic deviation from theoretical expectations, but this aspect needs further clarification.

The APA activity is pH dependent with a maximum in the slightly alkaline range. For activated sludge bound APA, an activity increase up to 30% was ascertained elevating the pH value from 7.00 to 8.50 [5]. Thus, to achieve comparable and reproducible results, the adjustment of a fixed and buffered pH value is necessary. According to the data of the studied wastewater treatment plant, the pH of the activated sludge spanned between 7.3 and 7.8 typically. Hence, we set the experimental pH to 7.5, stabilized by HEPES. There are several reasons for the application of this buffer. So far as known, this buffer interacts neither with the enzyme nor with solved metal ions tested as potential inhibitors. Also, it does not interfere with the fluorimetric analysis. Finally, its pK_a_ value of 7.55 fits very well with the desired working pH.

To gain data for proper modelling of the Michaelis-Menten function, initial substrate concentrations should span over two orders of magnitude at least and roughly one third of these concentrations should be smaller than the resulting K_M_ value. This requires some pre-experiments to get a coarse estimate of this half saturation constant. Based on the analysis of numerous sludge samples from the WWTP Trier, the apparent K_M_ value of the enzymatic hydrolysis of 4-MUF phosphate stretches from 10.0 to 30.0 µM/L mainly. Here, the term “apparent” accounts for the fact that neither the specific type or group of (iso-)enzyme(s) is known, contributing to the measured substrate hydrolysis, nor the influence of the reaction conditions, e.g., involvement of activators or inhibitors, or the importance of enzyme-sludge floc associations. A 4-MUF phosphate concentration range from 5.0 to 1200 µM/L has been proven to be feasible for APA kinetic measurements. Initial substrate concentrations below 5.0 µM/L are recommended in some cases, i.e., very low K_M_ values, but their practicability is restricted by the background fluorescence signal of the sludge suspension, compromising the reliability of the gained quantitative data.

Some aspects have to be considered selecting the assay time. Usually, Michaelis-Menten kinetics should be determined with data from the linear phase of the enzymatic reaction exclusively, where the reaction rates, as a function of the initial substrate concentration, are almost constant over time. Depending on the type of enzyme, on its catalytic activity for a given substrate under chosen test conditions and on the substrate : enzyme ratio, this period lasts from seconds to several minutes. To avoid running the reaction in the non-linear, substrate-depleting phase, one would prefer short assay times, e.g. one to two minutes. This procedure is suited studying pure microbial APA. Investigating the 4-MUF phosphate hydrolysis in HEPES buffer by pure microbial APA, concentration aligned to diluted activated sludge suspensions, the linear reaction period was less than 2 minutes for substrate concentrations ≤ 3.0 µM/L.

**Fig. 3:**
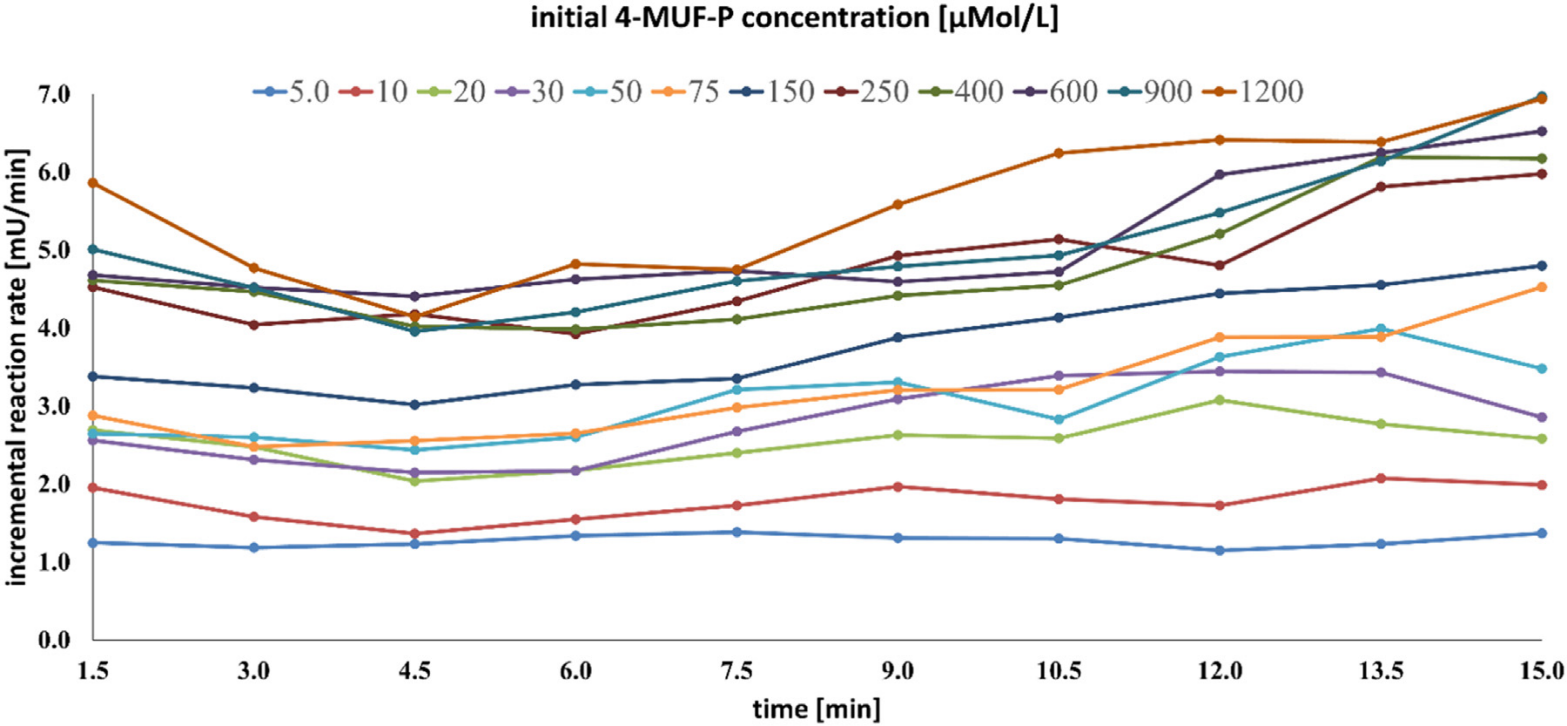
Example of incremental reaction rates for different substrate concentrations, calculated from APA standard kinetics. Test conditions: sludge sampling date: 2^nd^ March 2021, sludge predilution ratio 1:7.5, means of triplicates. Further experimental data: refer to Table 1.

Compared with the pure enzyme, the reaction profile of sludge bound APA is quite different. Depending on dosed substrate concentrations, the enzyme activity is below average during the first three to six minutes. At high substrate concentrations, the reaction rates tend to increase during ten minutes or more, whereas at low substance concentrations the rates remain more or less constant or tend to decrease. Consequently, an assay time of 15 – 20 min should offer a workable compromise. A plot of the rates, determined for every measuring interval (incremental rates) and every initial substrate concentration offers a useful control of the reaction course (Fig. 3).

Under ideal conditions (constant rates), a series of parallel straight lines would result, staggered from bottom to top according to increasing initial substrate concentration. Fig. 3 substantiates deviations from the ideal behavior. Minor variations of the incremental rates are discernible for transformations at low substrate concentrations. Stronger deviations are apparent at moderate and high 4-MUF-P concentrations. Here, the rates increase after roughly half of the reaction period.

**Fig. 4:**
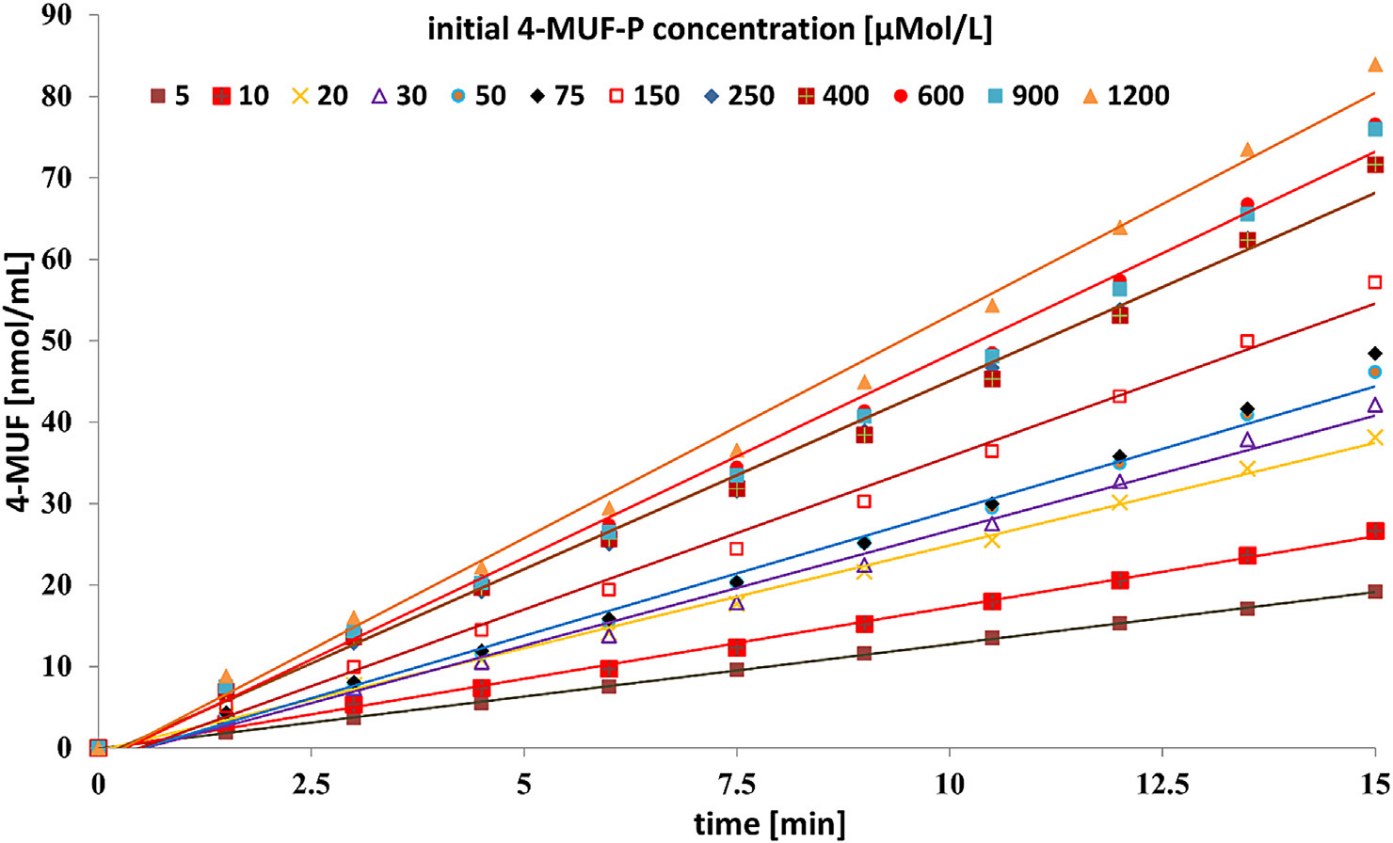
Linear regression functions of the concentration-dependent velocities of 4-MUF-P hydrolysis by activated sludge-bound APA. 4-MUF formation as a measure of the hydrolytical activity of APA. Reaction conditions as in Fig. 3.

The mean values of the concentration-related incremental rates are close to the slopes of the respective linear regression lines, calculated for the whole reaction period. The graphical representations of the linearized kinetic functions of the enzymatic hydrolysis at different substrate concentrations are combined in Fig. 4. The related slopes of regression lines (SLR) are listed in Table 1. Especially the kinetics at low initial substrate concentrations exhibit a high degree of linearity, confirmed by high coefficients of determination and by low RSD values of the incremental rates (Table 1). The latter provides a complementary measure to detect deviations from linear reaction velocity. With a few exceptions, the sequence of the increase of these RSD values corresponds to the sequence of decreasing determination coefficients.

**Table 1:** APA standard kinetics – example of experimental and modelled data.

4-MUF-P [µM/L]	experimental data				modelled data			
	Mean RSD [%]	Max RSD [%]	SDeg [%]	SLR [mU/mL]	CI [mU/mL]	r^2^	SER	RSD [%] incr. rates
5	11.1	14.2	25.5	1.281	± 0.016	0.9997	0.112	5.8
10	7.1	10.5	17.8	1.747	± 0.065	0.9975	0.456	12.2
20	7.7	9.7	12.7	2.520	± 0.094	0.9976	0.655	11.0
30	6.7	12.9	9.4	2.822	± 0.192	0.9919	1.334	17.4
50	9.8	11.2	6.1	3.061	± 0.175	0.9943	1.220	16.2
75	3.5	4.8	4.3	3.151	± 0.228	0.9909	1.583	19.8
150	8.1	9.5	2.5	3.754	± 0.233	0.9933	1.618	15.9
250	17.8	22.2	1.9	4.684	± 0.236	0.9956	1.639	14.1
400	12.4	14.2	1.2	4.619	± 0.265	0.9943	1.840	16.3
600	16.0	22.6	0.9	4.986	± 0.259	0.9953	1.799	15.0
900	16.1	23.3	0.6	4.892	± 0.288	0.9940	2.000	17.1
1200	3.7	5.2	0.5	5.466	± 0.318	0.9941	2.213	15.7

Experimental data: 10 groups of triplicates (one group per measuring time) per 4-MUF-P concentration; SDeg: percentage 4-MUF-P degradation after 15 min; SLR: slope of linear regression (kinetics); CI: confidence interval of SLR at 95% confidence level; r^2^: coefficient of determination; SER: standard error of regression. Experimental conditions: activated sludge sampling date 2^nd^ March 2021; sludge : buffer predilution ratio 1 : 7.5; assay time 15 min.

The graphical data representation in Fig. 4 suggests that some reactions, especially at higher substrate concentrations, run through two phases, i.e., a first phase with lower velocity, switching at around 7.5 min in a second phase with elevated velocity. In contrast to theoretical considerations, the comparably high percentage substrate degradation at low substrate concentration did not impair the linearity of the reaction progress. The high linearity of the kinetic functions (r^2^ > 0.99 always) fulfils one of the prerequisites for a sound fit to the Michaelis Menten kinetic model. A second criterion is the increase of the reaction rates with increasing substrate concentration until substrate saturation of the enzyme. This criterion is not completely fulfilled (invers relation at two positions within the data series). Nevertheless, these deviations are comparably small with respect to the presumably remaining heterogeneity of the sludge aliquots. The capability of a detailed analysis of reaction kinetics with a sufficiently high number of replicates is one of the most important benefits of the fluorimetric assay, which enables the quasi-continuous monitoring of the reaction course. Most photometric assays, often based on the determination of enzymatically released 4-nitrophenol from labelled substrates, require a reaction stop until measurement and, therefore, offer single point determination only [1].

Some aspects of the inhibition kinetic tests have to be mentioned too. To be able to develop a hypothesis regarding the inhibition mechanism, three inhibitor concentrations should be selected for discrete test series at least. For their proper selection, results from some pre-tests should be available. The knowledge of the inhibitor concentration, causing a 50% depression of the APA activity (IC_50_ value) at sludge-relevant APA concentration, would be advantageous. This parameter can be deduced from dose-response functions, where the 4-MUF-P concentration is fixed, and the inhibitor concentration is varied over 2 – 3 orders of magnitude [5].

Investigating oxyanions of transition metals as potential inhibitors, one has to take into account that solubility, ion charge, and molecular species are pH dependent. Exceeding compound specific concentration threshold values, they tend to form polyanions in the circumneutral and slightly alkaline pH range. To minimize the formation of polyanions, maximal concentrations of the monomeric oxyanions should not exceed 100 µM/L.

The sequence of the addition of the substrate and of the inhibitor can influence the reaction as well. According to our scheme, the inhibitor is added first, allowing for potential associations or reactions with the enzyme during the 10 min equilibration interval. Other protocols describe the simultaneous dosage of both components [6]. Whether it really makes a difference has to be tested and might depend on the prevailing inhibition mechanism.

**Fig. 5:**
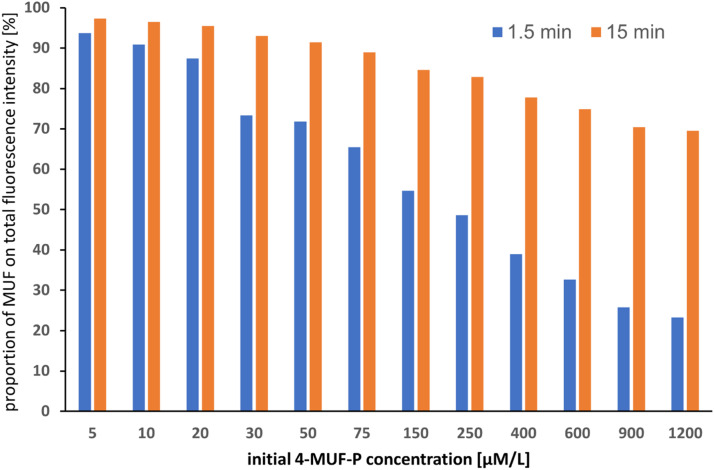
Proportion of the fluorescence emission of the enzymatically released 4-MUF on the total fluorescence signal at two times of measurement. Test conditions as in Fig. 3.

Some remarks to the analytical performance seem to be useful. To compensate for differences in the number and properties of sludge flocs transferred into the wells, kinetic tests are conducted as triplicates. Typically, the proportion of parallels with relative standard deviations of the fluorescence intensity exceeding 10% is less than one-tenth at a total of 132 readings of triplicates. There is a certain tendency of an increase of the relative standard deviations of the fluorescence readings with the increase of the 4-MUF-P concentrations, presumably caused by the concomitant increase of the proportion of the signal of the unhydrolyzed substrate (“negative control”) on the total fluorescence emission (Fig. 5). In the case of the exemplarily chosen kinetic investigation from 2^nd^ March 2021 (Table 1), the mean RSDs of the fluorescence emissions of the parallels with same initial substrate concentration span between 3.5 and roughly 18% with a clustering of higher RSDs at higher substrate concentrations.

4-MUF labelled substrates and 4-MUF as a quantitative indicator for the progress of an enzymatic hydrolysis reaction are in use since more than 50 years. Their analytical properties and benefits, i.e., high sensitivity and selectivity, are well documented [7,8]. Thus, discussion of analytical performance aspects can be focussed on some critical points with specific regard to the analysis of activated sludge.

Since the fluorescence emission of 4-MUF is pH- and temperature-dependent, the properties of the calibration standards and of the samples must match in this regard, i.e., the standards have to be constituted in 0.05 m HEPES (pH 7.50), too. The signal intensities of 4-MUF and 4-MUF phosphate decrease over time. The related rate depends on several factors, e.g., temperature and pH value. Usually, this effect is in sludge samples stronger than in sludge-free samples, which underlines the necessity of a matrix calibration. The high data acquisition rates of modern plate readers allow for a compensation of this type of sensitivity drift by the almost simultaneous reading of samples, negative controls, and calibration standards.

Assessing the analytical sensitivity, one has to take into account two background signals, i.e., the “negative control (NEG)” (4-MUF-P solved in HEPES buffer) and the calibration blank (activated sludge suspended in the buffer). The extremely low background signal of the pure buffer solution can be neglected. The background fluorescence of the blank varies within one order of magnitude at given sludge dilution, depending on the original sludge density and composition. Compared with the dimension of the relatively constant NEG signals, the blank signals correspond to NEGs with 10 – 30 µM/L of 4-MUF-P typically. The limit of quantitation (LOQ) is defined as the tenfold of the standard deviation of the NEG or the blank, whichever is higher, plus the absolute value of the NEG or the blank. Two consequences follow: i) The LOQ's of one test series are not constant but mainly NEG concentration specific. ii) At very low substrate concentration, the LOQ is defined by the reproducibility of the calibration blank signal. This LOQ criterion is relatively strong, since it integrates the time dependent decrease of the fluorescence signals. However, the resulting LOQs are usually surpassed at the first time of measurement for all initial substrate concentrations.

To deduce valid concentration values from low fluorescence signals, the appropriateness of the applied calibration range should be checked also. Even in cases where the calibration function offers a very high linearity, small deviations from linearity, often caused by a lower relative fluorescence intensity of the most concentrated standard, should not be neglected. Those deviations raise the ordinate intercept, leading to the calculation of lower analyte concentrations compared with calibration functions without ordinate intercept. Consequently, narrowing of the calibration range mediates this problem.

A critical point is the potential spectroscopic interference between 4-MUF and 4-MUF labelled substrates. For various combinations of them, a so-called “inner-filter” effect is described, where radiation absorption and emission of both components interacts with each other in a non-linear manner. In this case, corrections of the spectroscopic data are required [8,9]. Recently, Urvoy et al. have demonstrated that no inner-filter effect occurs in aqueous solutions of mixtures of 4-MUF and 4-MUF phosphate [10].

The combined fluorescence signal is always the sum of the emissions of both compounds, independently from their molar ratios and from their total amounts. Thus, it is justified to calculate the 4-MUF signal by subtraction of the 4-MUF phosphate signal (NEG) from the overall signal. As Fig. 5 reveals, the proportion of the substrate signal on the overall signal spans between roughly 3 and 80%, depending on initial 4-MUF phosphate concentration and on the reaction progress. Nevertheless, this manner of signal correction is afflicted with a systematic underestimation of the 4-MUF concentration since the substrate fluorescence is treated as constant despite of its decrease during the reaction. If the degree of substrate hydrolysis remains relatively low, the resulting error does not significantly influence the calculation of the kinetic parameters.

Finally, the accuracy of the method was checked by the addition of defined amounts of purified APA from *E. coli* (Invitrogen™, Cat. No. 18011015) with known activity to activated sludge samples and subsequent examination of the recovery of the added activity, i.e., corresponding increase of the maximal reaction velocity.

**Fig. 6:**
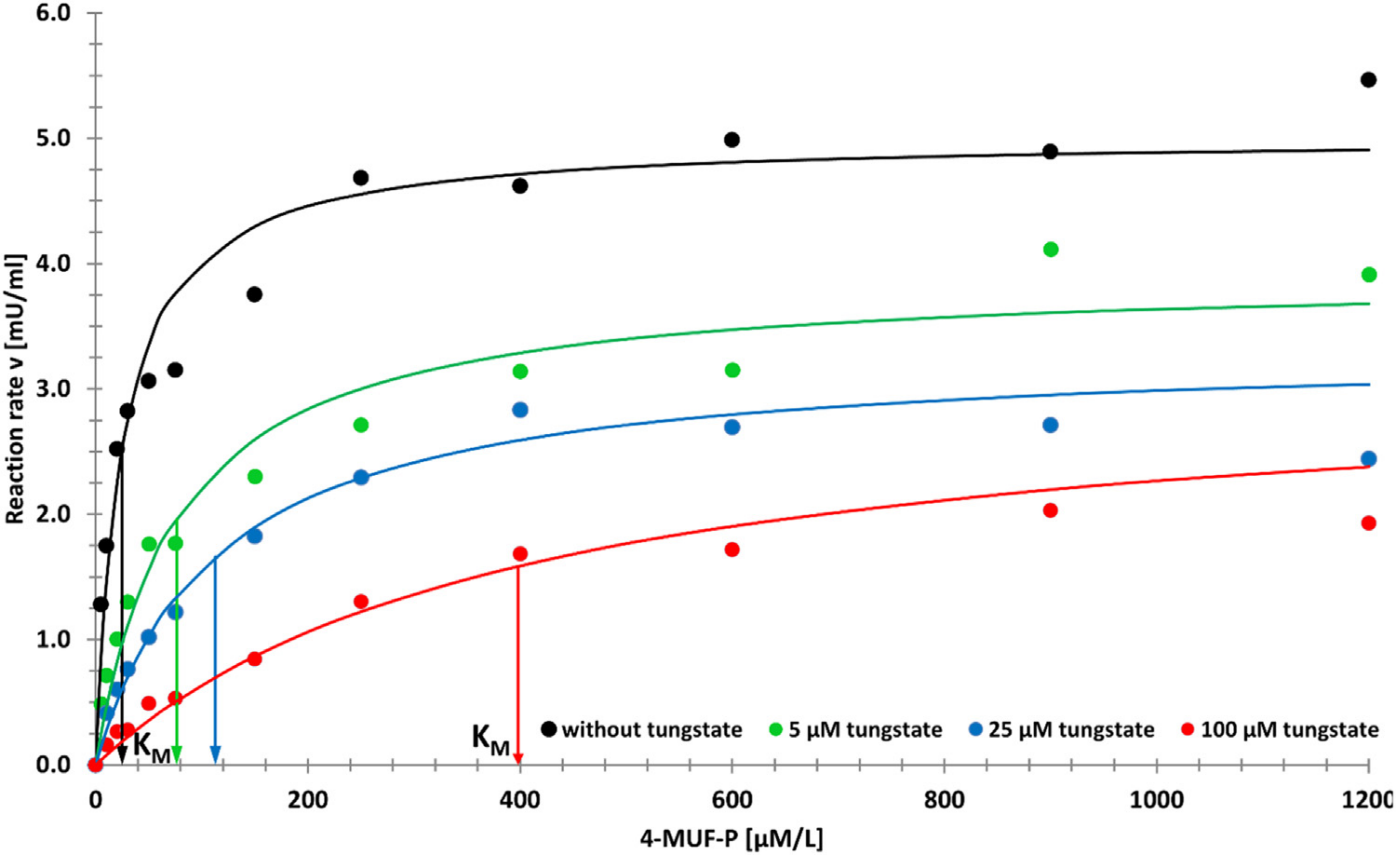
Michaelis Menten kinetics of the APA hydrolysis of 4-MUF-P with and without addition of tungstate as an inhibitor. Arrows indicate the K_M_ values. Reaction conditions as in Fig. 3 (from [11] with permission).

Fig. 6 displays an example of the application of the described method for the determination of the kinetics of APA inhibition by tungstate. The tests were conducted in the absence and presence (three different concentrations) of tungstate at pH 7.5. Essential results of the uninhibited kinetics were already documented in Fig. 3, Fig. 4, Fig. 5 and in Table 1. The most characteristic outcome of this study is the flattening of the curves with increasing inhibitor concentration. The calculated K_M_ values are listed together with further reaction figures in Table 2.

**Table 2:** APA inhibition by tungstate: essential experimental and modelling results.

WO_4_^2-^ [µM/L]	Triplicates**^a^** [%] RSD > 10 %	Single kinetics**^b^** r^2^ < 0.99 [n]	K_M_ [µM/L]	V_max_/ MMF**^c^** [mU/mL]	V_max_/ experimental [mU/mL]	SMSE**^d^** MMF
0	42	0	24.9	5.01	5.47	1.50
5	39	2	76.1	3.91	4.11	0.88
25	17	3	112.9	3.32	3.82	1.07
100	0	1	398.3	3.17	3.08	0.66

a) total of triplicates: 132; b) n_max_: 12; c) Michaelis Menten function; d) sum of mean square error Experimental conditions as in Table 1.

Inversely to the sequence of the K_M_ constants, the maximal reaction velocities decrease with increasing inhibitor concentrations. The differences between the experimental and modelled V_max_ values are comparably small. Interestingly, the proportion of replicates with higher RSDs decrease with increasing inhibitor concentration. The correlation between K_M_ value and inhibitor concentration is an indication for a competitive typ of inhibition, but the concomitant decrease of V_max_ points to additional side reactions.

## CRediT authorship contribution statement

**Klaus Fischer:** Conceptualization, Methodology, Formal analysis, Writing – original draft. **Marion Wacht:** Investigation.

## Declaration of Competing Interest

The authors declare that they have no known competing financial interests or personal relationships that could have appeared to influence the work reported in this paper.
